# Anti-inflammatory effects of resolvin-D1 on human corneal epithelial cells: *in vitro* study

**DOI:** 10.1186/1476-9255-11-6

**Published:** 2014-03-02

**Authors:** Nir Erdinest, Haim Ovadia, Ran Kormas, Abraham Solomon

**Affiliations:** 1 Department of Ophthalmology, Hadassah-Hebrew University Medical Center, Jerusalem, Israel; 2 Department of Neurology, The Agnes Ginges Center for Human Neurogenetics, Hadassah-Hebrew University Medical Center, Jerusalem, Israel; 3 Cornea & Refractive Surgery Service, Department of Ophthalmology, Hadassah-Hebrew University Medical Center, Jerusalem 91120, Israel

**Keywords:** Resolvin-D1, Polyunsaturated fatty acids, Corneal epithelium, Anti-inflammatory, Cytokines, Cornea

## Abstract

**Background:**

This study evaluated the anti-inflammatory effects of Resolvin-D1 (RV-D1) and its mechanism of action in human corneal epithelial (HCE) cells.

**Methods:**

HCE cells were incubated with different concentrations of RV-D1 for different time periods. Oleic acid (OA) and Dexamethasone (DM) served as negative and positive controls, respectively. Cells were stimulated with polyriboinosinic:polyribocytidylic acids (poly I:C). The protein contents and mRNA expression levels of Tumor necrosis factor-α (TNF-α), Interleukin (IL)-6, IL-1β and IL-8 were evaluated with multiplex fluorescent bead immunoassay (FBI) and real time-PCR, respectively. In addition, the expression of inhibitory factor-κBα (I-κBα) was evaluated with real time-PCR.

**Results:**

The protein level of pro-inflammatory cytokines TNF-α, IL-6, IL-1β and IL-8 significantly increased after stimulation with Poly I:C. RV-D1 treatment at concentration of 1 μM decreased the protein level of TNF-α to 20.76 ± 9.3% (P < 0.05), IL-6 to 43.54 ± 14.16% (P < 0.001), IL-1β to 46.73 ± 15.93% (P > 0.05) and IL-8 to 51.15 ± 13.01% (P < 0.05) compared with cells stimulated with poly I:C alone. Similarly, the mRNA levels of TNF-α, IL-6, IL-1β and IL-8 were significantly reduced after treatment with RV-D1. A highly significant dose response curve was demonstrated for RV-D1 treated HCE cells for TNF-α and IL-1β.

DM treatment decreased the protein content for all of the pro-inflammatory cytokines, similar results were demonstrated at the mRNA level. The anti-inflammatory effects of RV-D1 were similar to those of DM for TNF-α, IL-6 and IL-8.

**Conclusions:**

RV-D1 may serve as a potent anti-inflammatory agent in ocular surface inflammation, as evaluated in cultured HCE cells. The anti-inflammatory effects of RV-D1 were comparable to those of DM, and were mediated through nuclear factor kappa B (NF-κB) signal transduction.

## Background

Chronic inflammation of the ocular surface is a common characteristic of many common diseases such as the dry eye syndrome (DES), allergic conjunctivitis and contact lens intolerance [1-3]. The inflammatory process can cause significant damage to the corneal and conjunctival epithelia, with subsequent visual loss [4].

Current topical treatment options for ocular surface inflammation include mainly corticosteroids, non-steroidal anti-inflammatory agents and Cyclosporine A. Topical corticosteroids have shown efficacy in treating DES with a rapid onset of action [5], but are limited because of their potential side effect, such as posterior sub-capsular cataract and increased intraocular pressure [6]. Topical non-steroidal anti-inflammatory agents have limited anti-inflammatory activity and may impede wound healing [7]. Cyclosporin A takes a few weeks before achieving significant results, its efficacy is controversial, and it is indicated for mild to moderate inflammatory conditions [8].

Recently, there has been a large amount of interest in using poly-unsaturated fatty acids (PUFAs) as a treatment for ocular surface inflammatory disease [9]. A recent study has indicated that dietary or oral supplementation of PUFA may improve symptoms of DES and contact lens intolerance [10]. Dietary supplementation of Eicosapentaenoic acid (EPA) and Docosahexaenoic acid (DHA) reduced the prostaglandin levels in the dry eye rat model, and prevented the decrease in mucin production, an important symptom associated with DES [11]. A study by Rashid et al. using a dry eye mouse model has shown that topical administration of omega-3 fatty acids decreased dry eye signs and inflammatory changes at both cellular and molecular levels [12].

We have recently shown that alpha-linolenic acid (ALA), an omega-3 poly-unsaturated fatty acid, has potent anti-inflammatory effects on stimulated human corneal epithelial cells. Both protein and mRNA levels of several pro-inflammatory cytokines were dramatically decreased following treatment with ALA [13]. The results of that study have paved the way for further search of PUFAs bioactive derivatives, which may have a stronger anti-inflammatory effect.

Recently studies demonstrated that several of the bioactive derivatives of PUFAs possess potent anti-inflammatory effects, and can also promote the resolution of acute or chronic inflammatory processes. These studies have demonstrated a tightly controlled and programmed mechanism involving the resolution of inflammatory processes [14]. In order for an inflammatory process to be extinguished, the production of pro-inflammatory mediators should decrease, while the production of anti-inflammatory pro-resolution mediators should increase. In the PUFAs family, four new groups of such pro-resolution mediators have been identified: Lipoxins (LXs), Protectins (PTs), Maresins (MaR) and Resolvins (RVs). RVs are non-classical Eicosanoids, which are endogenous, potent, local acting molecules [4,14].

RVs can be formed by metabolizing EPA and DHA. Two main classes of resolvins have been identified. The E-series RVs are formed when EPA is metabolized first by cyclooxygenase-2 (COX2) or the cytochrome-P450 pathway in vascular endothelial cells, and then by neutrophil 5-lipooxygenase (5-LOX) [15]. On the other hand, the D-series RVs can be formed by metabolizing DHA first by 15-lipooxygenase (15-LOX) and then by 5-LOX. Another pathway to form the D-series RVs is based on the presence of Aspirin, which triggers COX-2 to metabolize DHA, which in turn is metabolized by 5-LOX to create RV-D [15].

Resolvins were shown to be extremely potent anti-inflammatory agents. Nano-molar doses of RV-E1 promote the resolution of inflammatory allergic airway responses by directly suppressing the production of IL-23 and IL-6, and inducing dramatic reduction of dermal inflammation, peritonitis, dendritic cell migration and inflammatory cytokine production [16,17]. RVs also limit polymorphic nuclear leukocytes infiltration and promote macrophages activity at the inflammation site, without causing systemic immunosuppressive activity [18].

Bazan et al’s study showed that after exposing mice to desiccating conditions, RV-E1 increased tear production by 60% compared to control [19]. Another study held by Pflugfelder et al. in a murine model of dry eye has shown that RV-E1 improves the outcome measures of corneal staining and goblet cell density, implying the potential of RVs as a future treatment for DES [20].

Resolvin D1 (RV-D1) is one of the mediators of the RV family, with potential anti-inflammatory effects. As of today, the direct local anti-inflammatory pro-resolution effects of RV-D1 on ocular surface cells have not been studied. In this study we investigated the direct local anti-inflammatory effects of RV-D1 on HCE cells in-vitro, comparing these effects with those of corticosteroids, in order to evaluate its efficacy as a possible anti-inflammatory agent for the treatment of ocular surface inflammatory disorders.

## Methods

The Hadassah Medical Center Institutional Review Board (IRB) approval was obtained for this study (IRB protocol number and version: EFA-EFE-IV-01), and all of the study procedures were carried out in accordance with the IRB guidelines. This study followed the tenets of the Declaration of Helsinki.

### Culture of Human Corneal Epithelial (HCE) cells

HCE cells were cultured from human corneoscleral rim explants, taken from several different human donors, provided by the Department of Ophthalmology at the Hadassah Medical Center, using a previously described method [21]. In brief, HCE cells were cultured in supplemented hormonal epithelial medium (SHEM) [22]. HCE cells were incubated at 37°C under 95% humidity and 5% CO_2_. The culture medium was replaced every other day. Cultures were kept for 10 to 14 days until a density of 90% confluence was observed. At this time, cells were passaged and seeded onto 6-well plates at a density of 2.0×10^5^ cells/well. Cells were observed by phase-contrast microscopy to ensure uniformity of morphology. The purity of HCE cultures was confirmed by staining for cytokeratin-19 with the indirect immune-peroxidase procedure with monoclonal antibody to human cytokeratin-19 (Abcam, Cambridge, UK). Second generation cells were used in all experiments.

### Drug preparation

Stock solution of RV-D1 was obtained by Cayman Chemical (Ann Arbor, Michigan, USA). Drug preparation and aliquots was performed at a nitrogen chamber, filtered through 0.2-μm-pore-size filters, divided to aliquots and sealed under nitrogen in opaque Eppendorf tubes. RV-D1 was stored at -80°C for no longer than 14 days before treatments.

### Experimental design

HCE cells were seeded into 6-wells plates for 24–48 hours before the experiment, at a density of 1.2 × 10^5^ cells/well in 2.0 ml of medium. Culture medium was exchanged every other day, and cultures were maintained until sub-confluence. RV-D1 was conjugated with Bovine serum albumin (BSA; Fraction V, Mercury, Israel) at a maximal concentration of 0.1% according to a previous study [23]. HCE cells were pre-incubated for two hours with RV-D1 before the inflammatory stimulus, based on a previous protocol [24]. In order to avoid oxidative effects, the fatty acids were defrosted once and were not reused again.

After incubation of the HCE cells with the RV-D1 and the controls, the cells were not washed out and were treated with Poly I:C at a dose of 25 μg/ml as previously described [25]. In addition, in order to examine the RV-D1 effect on baseline cytokines level, HCE cells were incubated without any stimulus, and then with and without RV-D1 treatments.

For maximal induction of IL-6 and IL-8, the stimulus exposure time lasted 4 hours for protein contents and 3 hours for mRNA expression levels, after the cells were pre-incubated for 2 hours with RV-D1 and the controls (hence, the total incubation time was 6 and 5 hours for protein and mRNA, respectively). For TNF-α and IL-1β, the stimulus lasted 15 hours for protein contents, and 12 hours for mRNA expression levels, after cells were pre-incubated for 2 hours with the RV-D1 and the controls (therefore, the total incubation time was 17 and 14 hours for protein and mRNA, respectively). In addition, RV-D1 was incubated at doses of 1 μM, 0.1 μM and 0.01 μM for dose–response curves evaluation.

Dexamethasone (DM) served as a positive control at a concentration of 10^−5^ M [26], while Oleic acid (OA) served as a negative control, at a concentration of 200 μM, due to its lack of effect on eicosanoid biosynthesis [27]. At the end of each treatment, the cells were examined with FITC-Annexin V/PI. Culture supernatants were collected, aliquoted and stored at -80°C for further measurements of the protein contents of the inflammatory mediators. Total RNA was extracted from the cells and stored at -80°C until thawed for cDNA-PCR.

Two hours before the experiment, the medium was replaced to 2 ml of serum-free medium (SFM) in each well. All the study experiments were performed in HCE cells that were cultured from human corneoscleral rim explants, taken from four different human donors.

### HCE cells viability

The apoptosis assay was performed as described previously [28]. In brief, at the end of each treatment HCE cells viability was assessed by flow cytometry using an Annexin V apoptosis detection kit (MBL, Nagoya, Japan). One μg/ml of Annexin V-FITC and 1 μg/ml of Propidium iodide (PI) were added to the cell suspension, and the mixture was incubated in the dark for 5 min at room temperature. Without washing, the cells were placed in 500 μl of Annexin V binding buffer and kept on ice, and within 5 min were evaluated using a Coulter FC-500 flow cytometer (Beckman-Coulter, Fullerton, CA, USA). Flow cytometry data were analyzed using CXP Analysis 2.0 software (Beckman-Coulter, Miami, FL, USA).

### Protein contents of inflammatory cytokines - multiplex Fluorescent Bead Immunoassay (FBI)

The cytokines protein concentration levels were measured using a multiplex fluorescent bead immunoassay (Cytometric bead array, CBA, Human Inflammatory Cytokines Kit, BD Biosciences, San Jose, CA, USA). This kit allows the simultaneous measurement of protein levels of Interleukin (IL)-1β (IL-1β), IL-6, IL-8, IL-10, Tumor Necrosis Factor Alpha (TNF-α), and IL-12 (p70) in a single sample. The test was performed and analyzed according to the manufacturer’s instructions and was performed as described previously [29]. Briefly, 50 μl of six premixed capture beads populations (coated with capturing antibodies specific for different cytokines or chemokines) were mixed with 50 μl of the provided standards. Eight point standard curves ranging from 20 to 5000 pg/ml were obtained by serial dilutions of the reconstituted lyophilized standards. Fifty microliters of capture beads populations were also mixed with culture supernatant samples, allowing for the different cytokines in the samples to be captured by their analogous beads. Afterwards, the cytokines capture beads were mixed with 50 μl of phycoerythrin-conjugated detection antibodies to form sandwich complexes. These sandwich complexes were successively incubated in the dark for 3 hours at room temperature. After incubation the mixture was washed, centrifuged (at 200× g for 5 min) and the pellet was resuspended in 300 μl of wash buffer. The BD LSRII flow cytometer (BD Biosciences, San Diego, CA, USA) was calibrated with setup beads and 1,800 events were acquired for each sample. Results were generated in graphical format and analyzed with FCAP array software v1.0.1 (BD Biosciences, San Diego, CA, USA).

### RNA isolation

Total RNA was extracted from the HCE cell samples with RNAqueous Kit (Ambion, Austin, TX, USA) following the manufacturer’s instructions. Quantification of total RNA was performed in a NanoDrop spectrophotometer (ND-1000; Nano-Drop Technologies, Wilmington, DE, USA). RNAs were stored at −80°C until further utilization.

### cDNA synthesis

cDNA was synthesized from purified and concentrated 0.5 μg RNA from each sample using a High Capacity cDNA Reverse Transcription Kit (Applied Biosystems, ABI, USA). A 20 μl total reaction volume was made with 10 μl RNA, 2 μl 10× RT buffer, 0.8 μl dNTP Mix (100 mM), 2.0 μl 10× RT random hexamer primers, 1.0 μl MultiScribe™ reverse transcriptase, 1 μl RNase inhibitor and 3.2 μl nuclease-free water. Synthesis was carried out in an ABI 7900 Thermo cycler (Applied Biosystems, Foster City, CA, USA) and reaction conditions were 25°C for 10 minutes, 37°C for 120 minutes, and 85°C for 5 minutes. cDNA samples were stored at −20°C.

### Real-time polymerase chain reaction

Real-time polymerase chain reaction (PCR) was performed using TaqMan® Gene Expression Assays (Applied Biosystems, Foster City, CA, USA) in the ABI Prism 7900HT Sequence Detection System (Applied Biosystems, Foster City, CA, USA) as described previously [30]. Negative controls were included to evaluate DNA contamination of isolated RNA and reagents.

Real-time polymerase chain reaction assays for the selected cytokines were: TNF-α, IL-1β, IL-6 and IL-8 (Applied Biosystems, Foster City, CA, USA) and I-κBα (Applied Biosystems, Foster City, CA, USA). An amount of 1 μL cDNA was loaded in each total volume of 20 ml of reaction mixture and assays were performed in triplicates.

The fold changes of the gene expression in the samples were normalized to the endogenous gene hypoxanthine phosphoribosyltransferase 1 (HPRT1; Applied Biosystems, Foster City, CA, USA). Quantitative analysis was performed using the comparative (ΔΔC_T_) method, in which C_T_ value is defined as the cycle number in which the detected fluorescence exceeds the threshold value, ΔC_T_ is the difference between C_T_ of a target gene and the endogenous control, and ΔΔC_T_ is the difference between ΔC_T_ of the analyzed sample and the calibrator (control sample) [30]. The results were analyzed by DataAssist™ Software Version 2.0 (Applied Biosystems, Foster City, CA, USA).

### Statistical analysis

All tests were carried out on four independent cell cultures, derived from four different cornea donors, and performed in triplicates for each of the treatments. The levels of the cytokines protein contents and mRNA expression were calculated as a ratio relative to medium. Statistical analysis and multiple comparisons were performed by one-way ANOVA using the InStat software version 3.0 (GraphPad Software Inc, San Diego, CA, USA).

The median effective concentration (EC 50) of RV-D1 was calculated with sigmoid Emax model. The pharmacokinetic modeling was performed using WinNonlin® 6.2 software (Pharsight Corporation, USA).

## Results

### Cell viability following RV-D1 treatments

HCE cells were cultured with RV-D1 at several concentrations and periods of time as described above. Cell viability following RV-D1 and the control groups were examined with Annexin V-FITC PI assay (MBL, Nagoya, Japan) at the end of each treatment. The viability of the HCE cells at the end of the treatments was 95 ± 2% in comparison to the medium only, which served as a negative control (data not shown). There were no significant differences in cell viability between the treatment groups (RV-D1, OA, DM) and the negative control (medium only). In addition, there were no significant differences in cell viability among each concentration of the RV-D1 treatments (1 μM, 0.1 μM, 0.01 μM) and the negative control.

### Stimulation of HCE cells (multiplex FBI)

**Figure 1:**
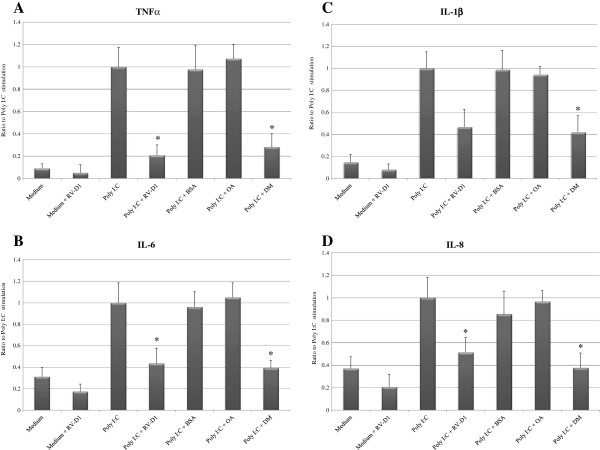
**RV-D1’s Effects on Poly I:C-Induced pro-inflammatory cytokines protein secretion in HCE cells (A-D).** For TNF-α and IL-1β detection the HCE cells were stimulated in SFM with Poly I:C (25 μg/cc) for 15 hours and cells pre-incubated for 2 hours with RV-D1 at 1 μM **(A, C)**. For IL-6 and IL-8 detection the HCE cells were stimulated with Poly I:C for 4 hours and cells pre-incubated for 2 hours with RV-D1 at 1 μM **(B, D)**. Data shown is the ratio to stimulus with poly I:C only (mean ± SD) from four independent experiments (n = 4). Asterisks indicate a significant difference (P < 0.05) between groups compared to poly I:C only.

HCE cells incubated with poly I:C expressed up to 4-fold higher levels of IL-6 (P < 0.05), 7-fold higher IL-1β (P < 0.01), 13-fold higher TNF-α (P < 0.01), and 3-fold higher IL-8 (P < 0.05) in HCE cells compared with incubation in medium only (Figures 1A-D).

### The RV-D1 effect on the baseline cytokines level

In order to examine the effect of RV-D1 on the background secretion level of the cytokines, RV-D1was incubated in un-stimulated cells (Figures 1A–D).

There were insignificant differences between the decrease of the cytokines (TNF-α, IL-6, IL-1β and IL-8) level after treatments with and without RV-D1 treatments in unstimulated cells.

### RV-D1 inhibit poly I:C-induced cytokines protein production (multiplex FBI)

RV-D1 treatment with concentration of 1 μM significantly decreased TNF-α protein secretion to 20.76 ± 9.3% (P < 0.05), IL-6 to 43.54 ± 14.16% (P < 0.001), IL-1β to 46.73 ± 15.93% (P > 0.05) and IL-8 to 51.15 ± 13.01% (P < 0.05), compared with poly I:C stimulus alone (Figures 1A–D). OA, which served as a negative control, did not cause a significant decrease in cytokines protein contents. These anti-inflammatory effects of RV-D1 were comparable with those of DM (excluding IL-1β), which decreased TNF-α protein secretion to 28.17 ± 11.90% (P < 0.05), IL-6 to 39.27 ± 6.96% (P < 0.001), IL-1β to 41.92 ± 15.0% (P < 0.05), and IL-8 to 37.63 ± 13.21% (P < 0.001), compared with incubation of HCE cells with poly I:C only. There was no significant difference between the decrease in cytokines level after treatments with either DM or RV-D1 in HCE cells after stimulation with poly I:C (excluding IL-1β), suggesting again similar anti-inflammatory effects of RV-D1 and DM.

### A dose–response reduction of cytokines by RV-D1 in poly I:C–stimulated HCE cells (examined by multiplex FBI)

HCE cells were further incubated with RV-D1 at concentrations of 1 μM, 0.1 μM and 0.01 μM respectively. For maximal induction of IL-6 and IL-8, the stimulus exposure time lasted 4 hours, and for TNF-α and IL-1β the stimulus lasted 15 hours.

**Figure 2:**
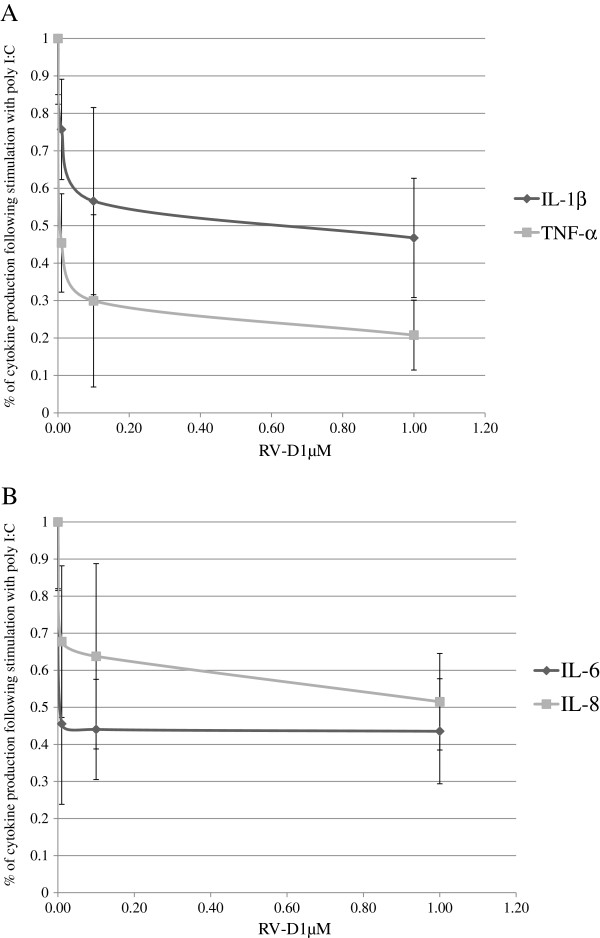
**A dose–response reduction of TNF-α, IL-1β (A) IL-6, IL-8 (B) by RV-D1 in Poly I:C (25 μg/cc) stimulated in HCE cells.** For TNF-α and IL-1β detection the HCE cells were incubated in SFM with RV-D1 for 15 hours and cells pre-incubated for 2 hours with RV-D1’s at 1 μM, 0.1 μM and 0.01 μM **(A)**. For IL-6 and IL-8 detection the HCE cells were incubated in SFM with Poly I:C for 4 hours and cells pre-incubated for 2 hours with RV-D1at 1 μM, 0.1 μM, and 0.01 μM **(B)**. For IL-1β and TNF-α the dose response correlation coefficients were >0.942 (p < 0.05).

Significant dose response curves were demonstrated for RV-D1 treatment doses for TNF-α and IL-1β (Figure 2A). The median effective concentration (EC 50) of RV-D1 values were 460.91 ± 280 nM for IL-1β and 4.81 ± 1.33 nM for TNF-α and the dose response correlation coefficients were >0.942 (p < 0.05). In contrast, there were insignificant differences in EC 50 of values for IL-6 and IL-8 (Figure 2B).

### Inhibition of Poly I:C-induced gene expression of cytokines examined by real-time PCR

**Figure 3:**
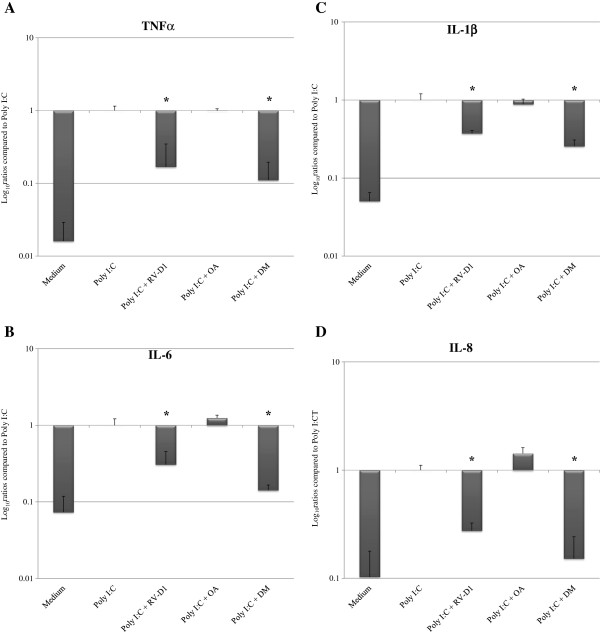
**RV-D1’s effects on poly I:C-induced cytokines mRNA expression levels in HCE cells, examined by real-time PCR (A–D).** mRNA expression levels are presented on a logarithmic scale. For TNF-α and IL-1β detection the HCE cells were incubated in SFM with RV-D1 for 12 hours and cells pre-incubated for 2 hours with RV-D1 at 1 μM **(A, C)**. For IL-6 and IL-8 detection the HCE cells were incubated in SFM with Poly I:C for 3 hours and cells pre-incubated for 2 hours with RV-D1’s at 1 μM **(B, D)**. Data is expressed as percentage (mean ± SD) of the expression found after poly I:C stimulation, which was normalized to 1.0, and derived from four independent experiments (n = 4). Asterisks indicate a significant difference (P < 0.05) between groups compared to poly I:C only.

The mRNA expression levels of IL-6, IL-1β, TNF-α, and IL-8 were significantly increased in HCE cells upon stimulation with poly I:C (Figures 3A–D), compared with un-stimulated cells. RV-D1 treatment in HCE cells after stimulation with poly I:C (Figures 3A–D) elicited a significant reduction of TNF-α mRNA levels to 16.81 ± 17.9% (p < 0.01), IL-6 to 30.67 ± 14.84% (p < 0.05), IL-1β to 37.26 ± 3.42% (p < 0.05), and IL-8 to 27.55 ± 4.95% (p < 0.01), compared with incubation of the cells with poly I:C only. DM treatment decreased TNF-α mRNA expression levels to 11.03 ± 8.5% (p < 0.01), IL-6 to 14.14 ± 2.42% (p < 0.01), IL-1β to 25.5 ± 5.26% (p < 0.01), and IL-8 to 15.22 ± 8.99% (p < 0.01), compared with incubation of the cells with poly I:C only.

There were insignificant differences (P > 0.05) observed in the levels of all the cytokines mRNA expression levels between the DMs treatment and RV-D1s treatment.

### Effects of RV-D1 on inhibitory factor-IκBα (I-κBα) mRNA expression levels

**Figure 4:**
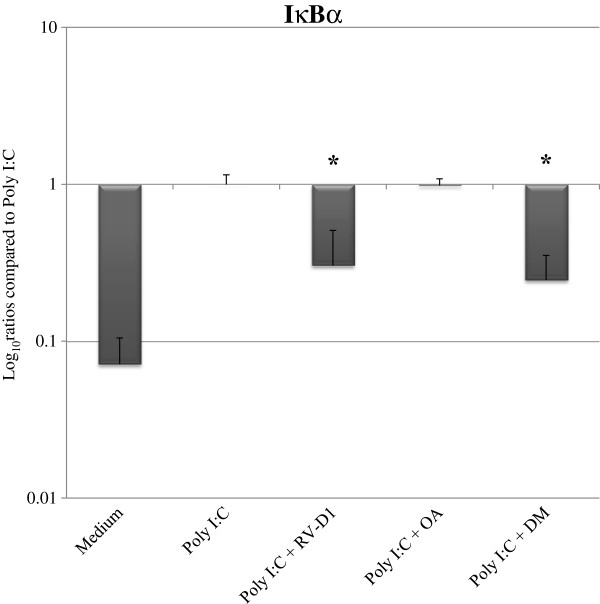
**RV-D1’s effects on Poly I:C-induced IκBα mRNA expression levels in HCE cells.** HCE cells were stimulated in SFM with Poly I:C (25 μg/cc) for 12 hours after cells pre-incubated for 2 hours with RV-D1’s 1 μM. mRNA expression levels of IκBα were examined by real time PCR. The mRNA expression levels are presented in a logarithmic scale, data are expressed as percentage (mean ± SD) of the Poly I:C stimulus which was normalized to 1.0 from three independent experiments (n = 3). Asterisks indicate a significant difference (P < 0.05) between groups compared to poly I:C only.

RV-D1 treatment in HCE cells after stimulation with poly I:C elicited a significant reduction of IκBa mRNA expression levels to 30.03 ± 20.51% (p < 0.05) (Figure 4), while DM treatment decreased IκBa mRNA expression levels to 24.6 ± 10.64% (p < 0.01) after stimulation with Poly I:C.

## Discussion

This study indicates a highly potent anti-inflammatory effect of RV-D1 on HCE cells in-vitro. RV-D1 reduced significantly the Poly I:C inflammatory reaction and dramatically reduced the production of the pro-inflammatory cytokines TNF-α, IL-6, IL-1β and IL-8 in cultured HCE cells. The reduction effect was demonstrated at both protein and gene expression levels for each one of these inflammatory mediators. A significant dose-dependent reduction was demonstrated by RV-D1 for TNF-α and IL-1β.

RV-D1 demonstrated inhibitory effects on the protein production of the inflammatory mediators, which were comparable to those of DM. Furthermore, the HCE cells stimulated by Poly I:C and treated with RV-D1 demonstrated a decrease in I-κBα mRNA expression, suggesting that the anti-inflammatory effects of RV-D1 involve regulatory effects of the nuclear factor kappa B (NF-κB) pathway.

Studies have shown an increase in the expression of pro-inflammatory mediators including adhesion molecules, chemokines and cytokines such as IL-1β, IL-6, IL-8, TNF-α in the tears and the ocular surface epithelia of DES patients and animal models [1,31]. Topical use of corticosteroids decreases the inflammatory response with a very high efficiency and a rapid onset of action [5]. Nevertheless, the disadvantages lay mainly in their significant adverse effects on long term use, as they may cause posterior sub-capsular cataract or increase the intraocular pressure [6,31]. Since most of the ocular conditions that involve inflammatory process, such as Sjögren’s syndrome and DES, are characterized by a chronic course, corticosteroids seem to have a limited role in their management. Another anti-inflammatory agent that has been tested topically is Cyclosporin A. The efficiency of this drug is controversial among different clinical researchers and studies. Apparently, Cyclosporin A has some efficiency, but it requires several months to obtain its maximal therapeutic anti-inflammatory effects, which can fit to the chronic course of these conditions [32]. Considering the significant disadvantages of topical corticosteroids treatment on one hand, and the limited efficiency of topical Cyclosporin A treatment on the other hand, there is still a necessity for an efficient yet safe agent for treating topically the chronic inflammatory diseases of the ocular surface.

During the recent years, a significant progress has been made on understanding the inflammatory process. It is now believed that an inflammatory process has 3 phases: onset, peak and resolution [14]. The onset is characterized by exceeded production of pro-inflammatory mediators, whereas during the peak there is a switch to the production of anti-inflammatory pro-resolution mediators, until their predominance divert the process towards resolution. Hence, an inflammatory process also triggers its own regulation, since as the production of anti-inflammatory pro-resolution mediators intensifies, the production of pro-inflammatory mediators decreases, and the inflammatory process is alleviated [14]. Studies which have evaluated the resolution phase have led to the understanding that anti-inflammatory pro-resolution agents can relieve the signs and symptoms of DES, and even lead to a complete resolution.

Studies which were done on the resolution phase had shown that the PUFAs bioactive derivatives have potent anti-inflammatory effects, qualities which give them a role at regulating acute or chronic inflammatory processes [14]. Rashid et al’s study has shown that the topical administration of omega-3 fatty acid in DES mouse model decreased the number of CD11b + cells and the levels of pro-inflammatory cytokines such as IL-1α and TNF-α in the ocular surface.^7^ Recently, a study we have conducted had shown that administration of Alpha linolenic acid (ALA) to HCE cells stimulated by Lipopolysaccharide (LPS) or polyriboinosinic:polyribocytidylic acids (poly I:C) in-vitro significantly reduced the protein and mRNA levels of several pro-inflammatory cytokines [13].

Realizing the potential PUFAs have, have led to extensive research of these materials, resulting in the identification of four new groups of molecules, which are metabolites of PUFAs: LXs (synthesized from arachidonic acid), RVs and PTs (derived from omega-3 fatty acids-EPA and DHA), and MaR (derived from DHA) [4,14]. Nowadays, tremendous efforts are invested in order to evaluate the anti-inflammatory pro-resolution impact of these bioactive derivatives on the inflammatory response.

RVs, as a family of bioactive PUFA derivates, have a vast range of actions, extracellular as well as intracellular. Their mechanism of action is not fully understood, but they seem to have effects on immune cells as well as non-immune ones, which can imply they have several biologic purposes. Studies have shown RVs decrease production of pro-inflammatory mediators as well as have immune-modulatory effects, which can imply on their anti-inflammatory pro-resolution activities [15-17].

RV-E1 has been studied for several years and may imply the potential anti-inflammatory effects of RV-D1, which is less studied. Recent studies have demonstrated administration of RV-E1 reduces neutrophils infiltration in murine models [33], can inhibit LTB4-stimulated transmigration in-vitro, and activate PMN phagocytosis in order to clean the tissue and reduce production of some pro-inflammatory cytokines such as IL-6 and INF-γ [15]. Because neutrophil infiltration is an early stage of any inflammatory process, and PMN phagocytosis is an early stage of resulotion [14,15], the fact that RVs reduce neutrophil infiltration by at least 4 different ways [15] and activate PMN phagocytosis, indicates they have a strong active pro-resolution effect.

A study of allergic airway inflammation indicates RV-E1 treatment reduces neutrophil and eosinophil infiltration, decreases Th2 number and decreases mucus production of the epithelium [34]. The result of the treatment was reduced lung damage and less bronchoconstriction [35]. Studies of colitis in a mice model have demonstrated that treating with RV-E1 reduced leukocytes amount, reduced production and secretion of IL-12, TNF-α and other pro-inflammatory cytokines [36] and decreased filled goblet cells [35]. The results were lower mortality rate, reduced weight loss and reduced colon shortening [36].

Recently, studies have investigated the effect of RV-E1 on different ocular pathologies. A study of corneal inflammation and neovascularization in a suture-induced model has demonstrated RV-E1 can reduce inflammation and neovascularization by dramatically minimizing leukocyte infiltration and reducing both TNF-α and VEGF-A production [37]. Furthermore, RV-E1 treatment has shown similar results comparing with traditional corticosteroids treatment in herpes simplex virus-mediated model of stromal keratitis [37].

In a mouse DES model, topical administration of RV-E1 has increased tear flow, promoted a healthy epithelium and decreased Cyclooxygenase-2 (COX2) expression and macrophage infiltration [19]. A unique feature of RVs is the added protection of host tissue by up-regulating pro-survival and down-regulating death pathways [19]. This effect is a major therapeutic goal in many inflammatory pathologies, including DES, since the barrier integrity of the tissue is damaged. These studies support the assumption that stimulating resolution pathways, such as RVs [15], could have a significant role in treating chronic inflammatory disorders, including DES.

A recent mice model of DES have shown that the treatment of RV-E1 reduces the corneal staining by 80% and the goblet density by 20% comparing to untreated controls, two major pathological expressions of DES [20]. Moreover, a study of DES in a mice model has shown treatment with RV-E1 may increase tear production by 60% and induce migration of superficial corneal epithelial cells to the dry areas [19].

RV-D1 has various effects on different organs as well, though apparently more effects are yet to be discovered. A recent study on acute lung injury in mice model showed reduced neutrophil infiltration and decreased production of TNF-α and IL-6 when treated with RV-D1 [38]. These effects were similar to those of RV-E1 [38]. A different study has demonstrated RV-D1 increases insulin sensitivity in adipose tissue [39]. A study of colitis in a mice model has shown RVs from the D-series can reduce production of pro-inflammatory cytokines such as TNF-α and IL-1β, similarly to the effects RV-E1 demonstrates in colitis [40]. Both E-series and D-series have shown a dramatic reduction of inflammatory pain in mice models, implying they have similar qualities [41].

Studies of ocular pathologies have focused mainly on RV-E1. Therefore, this line of treatment is more advanced. Recently, phase-II trials have been initiated for topical RV-E1 in patients with symptoms of DES [15]. Early results indicate symptomatic relief after a week under RV-E1 treatment comparing to placebo, and the relief was continued throughout the study period [15].

RV-D1 seems to have multiple sites of action, some overlap the ones RV-E1 activates, as mentioned above. The overlapping sites of action imply they may also have similar mechanism of action and therefore similar effect on several inflammatory pathologies, including inflammation of the ocular surface [42,43]. However, RV-D1 differs from RV-E1 not only by their molecular origin and molecular structures, but also by some features in their mechanism of action. Some examples for these differences are that RV-D1 does not activate ChemR23, a RV-E1 receptor [42]; moreover, the receptor of RvD1 has not been identified yet [43]. RvD1 significantly decreased PGE2 levels in choroid-retinal endothelial cells when stimulated with IL-1β, whereas RvE1 had little effect on PGE2 [43] and RV-D1 has no effect on intracellular Ca + 2 mobilization in neutrophils [15].

Only minor side effects from prolonged use with the systemic intake of n-3 PUFAs were described, mainly gastrointestinal distress [44], and no systemic side effects were described so far with the use of Resovlins, to the best of our knowledge.

Given that the study of RV-E1 is in advanced stages, together with the fact that RV-D1 has similar anti-inflammatory effects, but might also have added values due to the differences between the two mediators, we have decided to evaluate RV-D1 in our study.

The results of the study support previous studies which have shown that RVs are very potent [17,45]. Tian et al. found that RV-D1 and RV-E1 nano-molar concentrations treatment inhibit inflammatory signaling after choroid-retinal endothelial cells and leukocytes were stimulated with IL-1β [43]. The anti-inflammatory effects of RVs are more potent by a few orders of magnitude than the anti-inflammatory effects of ALA, which decreased pro-inflammatory cytokines in HCE cells following the treatment with micro-molar concentrations [13].

This study also showed that RV-D1 had inhibitory effects on Poly I:C induction, which caused a decrease in the I-κBα expression in HCE cells, suggesting decreased NF-κB activity. This finding is consistent with previous reports, which have shown the PUFA derivatives inhibit the LPS-induced NF-κB activation and subsequent TNF-α expression by decreased I-κBα phosphorylation and degradation [46]. In addition, recent in-vitro studies have shown that ALA has anti-inflammatory effects on HCE, which is mediated through NF-κB signal transduction [13], while n-3 PUFA inhibited NF-κB activation in an animal model of inflammation [47].

## Conclusions

Our study demonstrated several significant direct effects of RV-D1 in reducing inflammation of the ocular surface in-vitro. This is the first study to examine the anti-inflammatory effects of RV-D1 at the molecular level on a variety of cytokines which are involved in ocular surface inflammation and to assess its mechanism of action.

Altogether, our in-vitro work combined with the clinical data from studies on the systemic effects of orally administered RVs, demonstrate the significant anti-inflammatory effects of RV-D1. Since inflammation plays a crucial role in the pathogenesis of DES, the findings of this study have significant therapeutic implications; RV-D1 may be used as a potential topical anti-inflammatory treatment. Moreover, topical RV-D1 may be more beneficial than other current anti-inflammatory agents for ocular surface inflammation, which are limited by their undesired long term side effects.

Further clinical investigations are needed in order to assess the efficacy and safety of topical RV-D1treatment for ocular inflammation and DES in human subjects.

## Abbreviations

RV-D1: Resolvin-D1; HCE: Human corneal epithelial; OA: Oleic acid; DM: Dexamethasone; Poly I C: Polyriboinosinic polyribocytidylic acids; TNF-α: Tumor necrosis factor-α; IL: Interleukin; FBI: Fluorescent bead immunoassay; I-κBα: Inhibitory factor-κBα.

## Competing interests

The authors declare that they have no competing interests.

## Authors’ contributions

NE designed the study, performed the experiments, analyzed the data and wrote the manuscript. HO designed the study, performed the experiments, wrote the manuscript, analyzed the data and statistics wrote the manuscript. RK performed several experiments and analyzed data and statistics. AS designed the study, performed the experiments, analyzed the data and wrote the manuscript. All the experiments were performed in AS’s laboratory. All authors read and approved the final manuscript.

## Authors’ information

No conflicting relationship exists for any author.

## References

[B1] Dana MR Hamrah P Role of immunity and inflammation in corneal and ocular surface disease associated with dry eye Adv Exp Med Biol 2002 506 Pt B 729 738 12613985 10.1007/978-1-4615-0717-8_103

[B2] Thakur A Willcox MD Contact lens wear alters the production of certain inflammatory mediators in tears Exp Eye Res 2000 70 3 255 259 10.1006/exer.1999.0767 10712811

[B3] Lemp MA Contact lenses and allergy Curr Opin Allergy Clin Immunol 2008 8 5 457 460 10.1097/ACI.0b013e32830e6adc 18769201

[B4] Cortina MS Bazan HE Docosahexaenoic acid, protectins and dry eye Curr Opin Clin Nutr Metab Care 2011 14 2 132 137 10.1097/MCO.0b013e328342bb1a 21157308 PMC3971926

[B5] Marsh P Pflugfelder SC Topical nonpreserved methylprednisolone therapy for keratoconjunctivitis sicca in Sjogren syndrome Ophthalmology 1999 106 4 811 816 10.1016/S0161-6420(99)90171-9 10201607

[B6] Urban RC Jr Cotlier E Corticosteroid-induced cataracts Surv Ophthalmol 1986 31 2 102 110 10.1016/0039-6257(86)90077-9 3541262

[B7] Kim SJ Flach AJ Jampol LM Nonsteroidal anti-inflammatory drugs in ophthalmology Surv Ophthalmol 2010 55 2 108 133 10.1016/j.survophthal.2009.07.005 20159228

[B8] Schrell C Cursiefen C Kruse F Jacobi C Topical cyclosporine A 0.05% in the treatment of keratoconjunctivitis sicca Klin Monbl Augenheilkd 2012 229 5 548 553 22189827 10.1055/s-0031-1281862

[B9] Rand AL Asbell PA Nutritional supplements for dry eye syndrome Curr Opin Ophthalmol 2011 22 4 279 282 10.1097/ICU.0b013e3283477d23 21597374 PMC3155845

[B10] Kokke KH Morris JA Lawrenson JG Oral omega-6 essential fatty acid treatment in contact lens associated dry eye Cont Lens Anterior Eye 2008 31 3 141 146 10.1016/j.clae.2007.12.001 18313350

[B11] Viau S Maire MA Pasquis B Gregoire S Acar N Bron AM Bretillon L Creuzot-Garcher CP Joffre C Efficacy of a 2-month dietary supplementation with polyunsaturated fatty acids in dry eye induced by scopolamine in a rat model Graefes Arch Clin Exp Ophthalmol 2009 247 8 1039 1050 10.1007/s00417-009-1080-z 19415319

[B12] Rashid S Jin Y Ecoiffier T Barabino S Schaumberg DA Dana MR Topical omega-3 and omega-6 fatty acids for treatment of dry eye Arch Ophthalmol 2008 126 2 219 225 10.1001/archophthalmol.2007.61 18268213

[B13] Erdinest N Shmueli O Grossman Y Ovadia H Solomon A Anti-inflammatory effects of alpha linolenic Acid on human corneal epithelial cells Invest Ophthalmol Vis Sci 2012 53 8 4396 4406 10.1167/iovs.12-9724 22669722

[B14] Serhan CN Brain SD Buckley CD Gilroy DW Haslett C O’Neill LA Perretti M Rossi AG Wallace JL Resolution of inflammation: state of the art, definitions and terms FASEB J 2007 21 2 325 332 10.1096/fj.06-7227rev 17267386 PMC3119634

[B15] Zhang MJ Spite M Resolvins: anti-inflammatory and proresolving mediators derived from omega-3 polyunsaturated fatty acids Annu Rev Nutr 2012 21 32 203 227 22404117 10.1146/annurev-nutr-071811-150726

[B16] Ariel A Serhan CN Resolvins and protectins in the termination program of acute inflammation Trends Immunol 2007 28 4 176 83 10.1016/j.it.2007.02.007 17337246

[B17] Serhan CN Hong S Gronert K Colgan SP Devchand PR Mirick G Moussignac RL Resolvins: a family of bioactive products of omega-3 fatty acid transformation circuits initiated by aspirin treatment that counter proinflammation signals J Exp Med 2002 196 8 1025 37 10.1084/jem.20020760 12391014 PMC2194036

[B18] Serhan CN Novel lipid mediators and resolution mechanisms in acute inflammation: to resolve or not? Am J Pathol 2010 177 4 1576 91 10.2353/ajpath.2010.100322 20813960 PMC2947253

[B19] Li N He J Schwartz CE Gjorstrup P Bazan HE Resolvin E1 improves tear production and decreases inflammation in a dry eye mouse model J Ocul Pharmacol Ther 2010 26 5 431 9 10.1089/jop.2010.0019 20874497 PMC2956380

[B20] de Paiva CS Schwartz CE Gjorstrup P Pflugfelder SC Resolvin E1 (RX-10001) reduces corneal epithelial barrier disruption and protects against goblet cell loss in a murine model of dry eye Cornea 2012 31 11 1299 1303 10.1097/ICO.0b013e31823f789e 22257864

[B21] Li DQ Lokeshwar BL Solomon A Monroy D Ji Z Pflugfelder SC Regulation of MMP-9 production by human corneal epithelial cells Exp Eye Res 2001 73 4 449 59 10.1006/exer.2001.1054 11825017

[B22] Jumblatt MM Neufeld AH Beta-adrenergic and serotonergic responsiveness of rabbit corneal epithelial cells in culture Invest Ophthalmol Vis Sci 1983 24 8 1139 43 6135673

[B23] Pound EM Kang JX Leaf A Partitioning of polyunsaturated fatty acids, which prevent cardiac arrhythmias, into phospholipid cell membranes J Lipid Res 2001 42 3 346 51 11254745

[B24] De CR Cybulsky MI Clinton SK Gimbrone MA Jr Libby P The omega-3 fatty acid docosahexaenoate reduces cytokine-induced expression of proatherogenic and proinflammatory proteins in human endothelial cells Arterioscler Thromb 1994 14 11 1829 36 10.1161/01.ATV.14.11.1829 7524649

[B25] Ueta M Hamuro J Kiyono H Kinoshita S Triggering of TLR3 by polyI:C in human corneal epithelial cells to induce inflammatory cytokines Biochem Biophys Res Commun 2005 331 1 285 94 10.1016/j.bbrc.2005.02.196 15845391

[B26] Djalilian AR Nagineni CN Mahesh SP Smith JA Nussenblatt RB Hooks JJ Inhibition of inflammatory cytokine production in human corneal cells by dexamethasone, but not cyclosporin Cornea 2006 25 6 709 14 10.1097/01.ico.0000208815.02120.90 17077666

[B27] Knoch B Barnett MP Cooney J McNabb WC Barraclough D Laing W Roy NC Dietary oleic acid as a control fatty acid for polyunsaturated fatty acid intervention studies: a transcriptomics and proteomics investigation using interleukin-10 gene-deficient mice Biotechnol J 2010 5 11 1226 40 10.1002/biot.201000066 20872728

[B28] Vermes I Haanen C Steffens-Nakken H Reutelingsperger C A novel assay for apoptosis. Flow cytometric detection of phosphatidylserine expression on early apoptotic cells using fluorescein labelled Annexin V J Immunol Methods 1995 184 1 39 51 10.1016/0022-1759(95)00072-I 7622868

[B29] Vignali DA Multiplexed particle-based flow cytometric assays J Immunol Methods 2000 243 1–2 243 55 10986418 10.1016/s0022-1759(00)00238-6

[B30] Gibson UE Heid CA Williams PM A novel method for real time quantitative RT-PCR Genome Res 1996 6 10 995 1001 10.1101/gr.6.10.995 8908519

[B31] Javadi MA Feizi S Dry eye syndrome J Ophthalmic Vis Res 2011 6 3 192 8 22454735 PMC3306104

[B32] Donnenfeld E Pflugfelder SC Topical ophthalmic cyclosporine: pharmacology and clinical uses Surv Ophthalmol 2009 54 3 321 38 10.1016/j.survophthal.2009.02.002 19422961

[B33] Arita M Bianchini F Aliberti J Sher A Chiang N Hong S Yang R Petasis NA Serhan CN Stereochemical assignment, antiinflammatory properties, and receptor for the omega-3 lipid mediator resolvin E1 J Exp Med 2005 201 5 713 22 10.1084/jem.20042031 15753205 PMC2212834

[B34] Haworth O Cernadas M Yang R Serhan CN Levy BD Resolvin E1 regulates interleukin 23, interferon-gamma and lipoxin A4 to promote the resolution of allergic airway inflammation Nat Immunol 2008 9 8 873 9 10.1038/ni.1627 18568027 PMC2784998

[B35] Aoki H Hisada T Ishizuka T Utsugi M Kawata T Shimizu Y Okajima F Dobashi K Mori M Resolvin E1 dampens airway inflammation and hyperresponsiveness in a murine model of asthma Biochem Biophys Res Commun 2008 367 2 509 15 10.1016/j.bbrc.2008.01.012 18190790

[B36] Arita M Yoshida M Hong S Tjonahen E Glickman JN Petasis NA Blumberg RS Serhan CN Resolvin E1, an endogenous lipid mediator derived from omega-3 eicosapentaenoic acid, protects against 2,4,6-trinitrobenzene sulfonic acid-induced colitis Proc Natl Acad Sci USA 2005 102 21 7671 6 10.1073/pnas.0409271102 15890784 PMC1103706

[B37] Jin Y Arita M Zhang Q Saban DR Chauhan SK Chiang N Serhan CN Dana R Anti-angiogenesis effect of the novel anti-inflammatory and pro-resolving lipid mediators Invest Ophthalmol Vis Sci 2009 50 10 4743 52 10.1167/iovs.08-2462 19407006 PMC2763387

[B38] Wang B Gong X Wan JY Zhang L Zhang Z Li HZ Min S Resolvin D1 protects mice from LPS-induced acute lung injury Pulm Pharmacol Ther 2011 24 4 434 41 10.1016/j.pupt.2011.04.001 21501693

[B39] Hellmann J Tang Y Kosuri M Bhatnagar A Spite M Resolvin D1 decreases adipose tissue macrophage accumulation and improves insulin sensitivity in obese-diabetic mice FASEB J 2011 25 7 2399 407 10.1096/fj.10-178657 21478260 PMC3114534

[B40] Bento AF Claudino RF Dutra RC Marcon R Calixto JB Omega-3 fatty acid-derived mediators 17(R)-hydroxy docosahexaenoic acid, aspirin-triggered resolvin D1 and resolvin D2 prevent experimental colitis in mice J Immunol 2011 187 4 1957 69 10.4049/jimmunol.1101305 21724996

[B41] Xu ZZ Zhang L Liu T Park JY Berta T Yang R Serhan CN Ji RR Resolvins RvE1 and RvD1 attenuate inflammatory pain via central and peripheral actions Nat Med 2010 16 5 592 7 1p 10.1038/nm.2123 20383154 PMC2866054

[B42] Krishnamoorthy S Recchiuti A Chiang N Yacoubian S Lee CH Yang R Petasis NA Serhan CN Resolvin D1 binds human phagocytes with evidence for proresolving receptors Proc Natl Acad Sci USA 2010 107 4 1660 5 10.1073/pnas.0907342107 20080636 PMC2824371

[B43] Tian H Lu Y Sherwood AM Hongqian D Hong S Resolvins E1 and D1 in choroid-retinal endothelial cells and leukocytes: biosynthesis and mechanisms of anti-inflammatory actions Invest Ophthalmol Vis Sci 2009 50 8 3613 20 10.1167/iovs.08-3146 19443724

[B44] Hodge W Barnes D Schachter HM Pan Y Lowcock EC Zhang L Sampson M Morrison A Tran K Miguelez M Lewin G Effects of omega-3 fatty acids on eye health Evid Rep Technol Assess (Summ ) 2005 117 1 6 16111433 PMC4780934

[B45] Mukherjee PK Marcheselli VL Serhan CN Bazan NG Neuroprotectin D1: a docosahexaenoic acid-derived docosatriene protects human retinal pigment epithelial cells from oxidative stress Proc Natl Acad Sci USA 2004 101 22 8491 6 10.1073/pnas.0402531101 15152078 PMC420421

[B46] Zhao Y Joshi-Barve S Barve S Chen LH Eicosapentaenoic acid prevents LPS-induced TNF-alpha expression by preventing NF-kappaB activation J Am Coll Nutr 2004 23 1 71 8 10.1080/07315724.2004.10719345 14963056

[B47] Ohtsuka Y Okada K Yamakawa Y Ikuse T Baba Y Inage E Fujii T Izumi H Oshida K Nagata S Yamashiro Y Shimizu T Omega-3 fatty acids attenuate mucosal inflammation in premature rat pups J Pediatr Surg 2011 46 3 489 95 10.1016/j.jpedsurg.2010.07.032 21376198

